# Effect of single-day versus multi-day low-residue diet on colonoscopy bowel preparation: a systematic review and meta -analysis

**DOI:** 10.1186/s12876-025-04261-8

**Published:** 2025-09-26

**Authors:** Jing Du, Xueqian He, Peng Li

**Affiliations:** 1 Cancer Center, Department of Gastroenterology, Zhejiang Provincial People’s Hospital, Affiliated People’s Hospital, Hangzhou Medical College, Hangzhou, Zhejiang China; 2 Laboratory Medicine Center, Department of Clinical Laboratory, Zhejiang Provincial People’s Hospital, Affiliated People’s Hospital, Hangzhou Medical College, Hangzhou, Zhejiang China; 3 Department of Clinical Laboratory, Sir Run Run Shaw Hospital, Zhejiang University School of Medicine, Hangzhou, Zhejiang China; 4 158 Shangtang Road, Hangzhou, 310014 Zhejiang China

**Keywords:** Low-residue diet, Colonoscopy, Bowel preparation, Patient tolerance, Meta-analysis

## Abstract

Introduction: To compare the effects of single-day and multi-day low-residue diets on bowel preparation quality and patient tolerance of colonoscopy. Methods: A systematic review and meta-analysis of randomized controlled trials were conducted to compare the efficacy of a single-day low-residue diet and a multi-day low-residue diet for bowel preparation for colonoscopy. Relevant articles were screened from PubMed, Web of Science, Cochrane Library, and Embase databases from inception till December 10, 2024. Data were extracted and analyzed using Review Manager software after data extraction. Results: In total, 2248 patients from five randomized controlled trials were included in the meta-analysis. In the intention-to-treat population, the adequate bowel preparation rates were 90.1% and 89.7% in the single-day and multi-day low-residue diet groups, respectively (pooled odds ratio [OR] = 1.03, 95% confidence interval [CI]: 0.76–1.41, *p* = 0.84). In the per-protocol population, the adequate bowel preparation rates were 91.3% and 90.2% in the single-day and multi-day low-residue diet groups, respectively (pooled OR = 1.14, 95% CI: 0.84–1.54, *p* = 0.40). Four studies examined patient tolerance (pooled OR = 1.87, 95% CI: 1.52–2.31, *p* < 0.01), whereas three examined the patients’ willingness to repeat the same dietary restrictions (pooled OR = 2.91, 95% CI: 1.61–5.25, *p* < 0.01). Conclusions: There is no significant difference in the quality of bowel preparation between single-day and multi-day low-residue diets before colonoscopy. However, patient tolerance and willingness to repeat the same dietary restrictions were higher in the single-day low-residue diet than in the multi-day low-residue diet.

**Supplementary Information:**

The online version contains supplementary material available at 10.1186/s12876-025-04261-8.

## Introduction

Colonoscopy is an important endoscopic examination method for screening, diagnosing, and treating colorectal lesions [1]. The precision of colonoscopic diagnosis and the safety of therapeutic intervention are intimately related to bowel preparation quality [2]. Adequate bowel preparation is crucial for achieving high-quality colonoscopy [3]. Inadequate bowel preparation reduces the effectiveness and safety of colonoscopy and adenoma detection rate [4]. Risk factors for inadequate bowel preparation include chronic constipation, obesity, inadequate polyethylene glycol dosage, and a pre-endoscopic high-fiber diet [5]. Dietary restriction can significantly enhance the quality of intestinal preparation by diminishing the food residue in the gut, thereby increasing colon cleanliness [6]. Research has shown that compared with a clear liquid diet (CLD), a low-residue/low-fiber diet before colonoscopy improves patient tolerance for bowel preparation and increases their willingness to repeat a similar bowel preparation, without significant differences in the quality of bowel preparation or the incidence of adverse reactions [7]. A low-residue diet (LRD) is defined as a diet that restricts foods high in fiber and other indigestible substances that contribute to stool bulk, with the aim of reducing the frequency and volume of bowel movements. Thus, current guidelines for bowel preparation recommend a LRD before colonoscopy [8]. However, there is no consensus on the duration of the low-residue diet. Some studies used a 3-day low-residue diet prior to colonoscopy to improve bowel preparation quality [9]. Nevertheless, other studies have pointed out that > 1-day dietary restrictions prior to colonoscopy do not improve bowel preparation quality [10]. Moreover, a prolonged dietary restriction can affect patient tolerance [11].

Recently, a meta-analysis by Putri et al. [12] directly compared the bowel cleansing efficacy of single-day and multi-day LRD regimens. Their seminal work conclusively demonstrated that a single-day LRD is non-inferior to a multi-day regimen in terms of the quality of bowel preparation, as measured by standardized cleanliness scales. This finding represents a significant step forward in optimizing colonoscopy preparation protocols.However, the evaluation of a bowel preparation regimen cannot be solely confined to its cleansing efficacy. High-quality preparation is also intrinsically linked to patient tolerability and adherence. A regimen that is effective but poorly tolerated may lead to non-compliance, ultimately resulting in inadequate preparation and failed procedures. Therefore, the critical question remains: does the non-inferior efficacy of the single-day LRD translate into a superior patient experience without compromising clinical outcomes? To date, this patient-centered perspective has not been comprehensively evaluated in a systematic synthesis of the evidence. The existing meta-analysis by Putri et al. focused primarily on efficacy endpoints. Our study therefore aims to build upon this foundation by not only verifying the cleansing efficacy but also, and more importantly, by integrating patient-reported outcomes (PROs) such as tolerability, and willingness to repeat the regimen. This work is designed to provide a more holistic assessment, bridging the gap between biological efficacy and pragmatic effectiveness in clinical practice.

## Methods

This meta-analysis was performed according to the standards of the Preferred Reporting Items for Systematic Reviews and Meta-Analyses (PRISMA) [13].

### Inclusion and exclusion criteria

The inclusion criteria were: (1) randomized controlled trials on the effect of different duration (days) of low-residue diet on the quality of bowel preparation for colonoscopy; (2) the intervention group should be on a single-day low-residue diet, and the control group should be on a multi-day (≥ 2 days) low-residue diet; (3) the study participants were adult patients > 18 years old; (4) the primary outcome included the rate of adequate bowel preparation; (5) only English studies were included.

The exclusion criteria were: (1) cohort studies, case-controlled trials, and non-randomized controlled trials; (2) conference abstracts.

### Search strategy

Two researchers independently searched the following English databases for articles published from inception to December 10, 2024: PubMed, Web of Science, Cochrane Library, and Embase. The main English search terms were “low-residue diet,” “low-fiber diet,” “colonoscopy,” “bowel preparation,” and “colon cleansing.” First, the title and abstract of the studies were screened, and those that did not meet the inclusion criteria were excluded. Then, the remaining studies were downloaded, and the full text was read for screening them according to the inclusion and exclusion criteria. The title and abstract screening and full-text screening were conducted independently by two researchers, and discrepancies were resolved through discussion with a third reviewer. Lastly, the randomized controlled trials that met the inclusion criteria were included in this meta-analysis.

### Bias evaluation and data extraction

The included studies were evaluated for bias using the Cochrane risk-of-bias tool for randomized trials (RoB 2) [14]. The two researchers worked independently. In case of disagreements, discussion was undertaken to resolve the conflicts. If the disagreement persisted, a third researcher was consulted. The relevant data from the single-day and multi-day low-residue diet groups from the included studies were extracted, including the first author, publication year, study time, sample size, patient age, sex distribution, period of the low-residue diet, bowel preparation regimens, bowel preparation evaluation scale, definition of adequate bowel preparation, patient tolerance, and colonoscopy results.

### Primary and secondary outcomes

The primary outcome was the rate of adequate bowel preparation in the intention-to-treat (ITT) and per-protocol (PP) populations. The secondary outcomes were the patient tolerance, willingness to repeat similar dietary restrictions, adenoma detection rate (ADR), polyp detection rate (PDR), and adverse events or incidents. The patient tolerance was defined as less difficulty in or easily compliance to following the prescribed diet. The ADR and PDR were defined as the percentage of colonoscopies that detected at least one adenoma and one polyp, respectively.

### Statistical analysis

A meta-analysis was performed using the Review Manager software (version 5.3, the Cochrane Collaboration, Oxford, England). Dichotomous data were entered as the number of events and the total number of patients. The presence of heterogeneity was detected by the *χ*^2^ test, and the degree of heterogeneity was assessed by *I*^2^. Pooled analysis was performed with the random effects model due to the potential heterogeneity in included studies. Inverse variance was used as the statistical method for this meta-analysis. Dichotomous data were evaluated by odds ratio (OR) and 95% confidence interval (CI). A p-value of < 0.05 was considered statistically significant. Sensitivity analysis was performed by excluding one study at a time for the primary outcome. For outcomes with incompatible data formats, a meta-analysis was not performed. Instead, we conducted a synthesis without meta-analysis. This involved a vote count based on the direction of effect and a structured narrative synthesis to explore sources of heterogeneity and present findings transparently. A funnel plot was used to evaluate publication bias if more than 10 studies were included.

### Patient and public involvement statement

None.

## Results

### Literature search and study characteristics

**Fig. 1:**
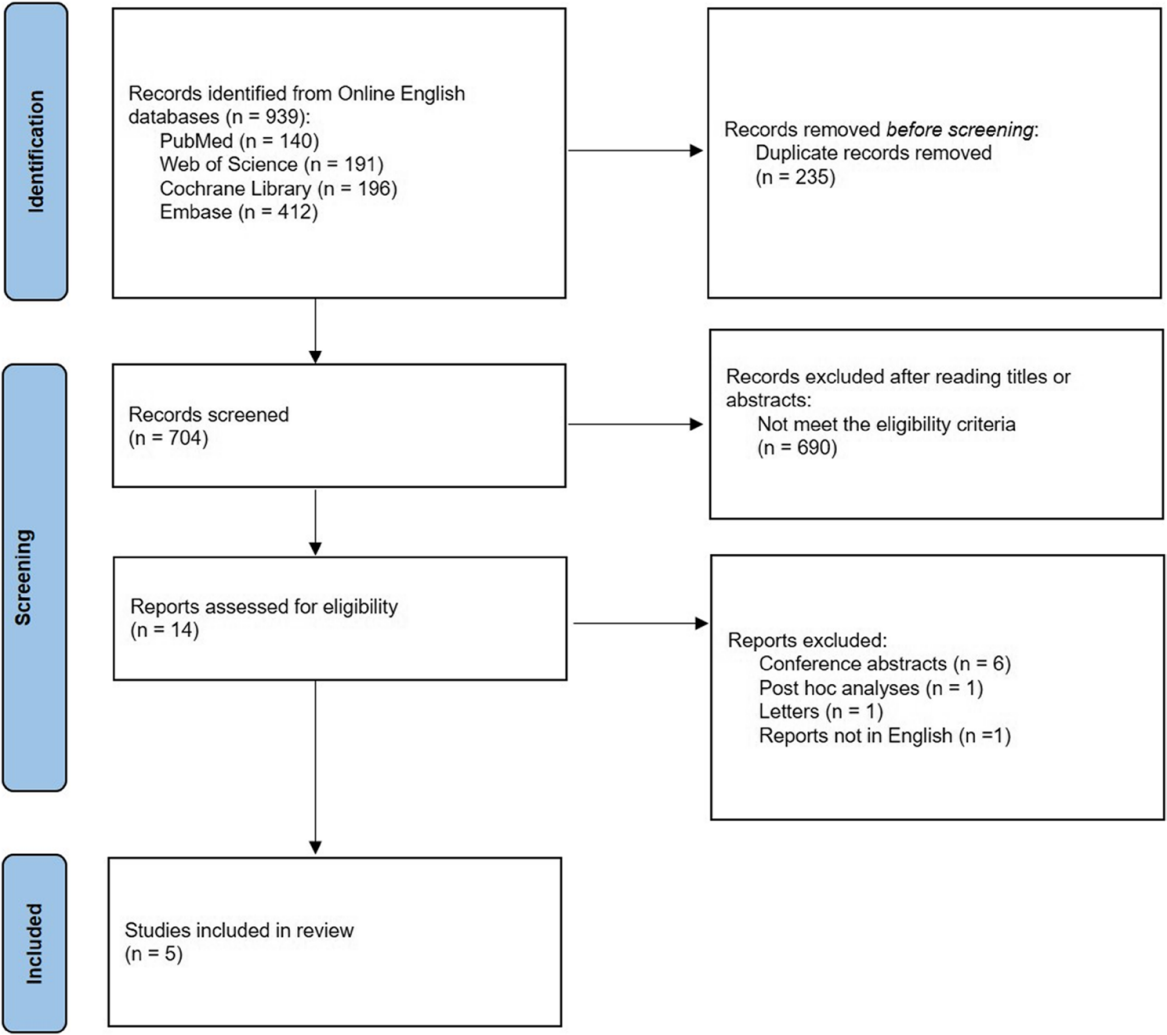
PRISMA flow diagram for studies included and excluded

A total of 1041 articles were obtained from online databases, and the full text of 14 articles was downloaded by screening the article titles and abstracts. Five randomized controlled trials [15–19] comprising 2248 patients were finally included in the meta-analysis after the application of the inclusion and exclusion criteria (Fig. 1). Four studies [15, 17–19] were conducted in Europe and compared 1-day and 3-day low-residue diets, whereas one study [16] was conducted in China and compared 1-day and 2-day low-residue diets. Four included studies [15, 16, 18, 19] recruited adult participants undergoing screening, surveillance, and diagnostic colonoscopy. The other [17] involved participants from an early colorectal cancer detection program aged 50–69 years, with positive fecal immunochemical test results. All studies used the Boston Bowel Preparation Scale (BBPS) to evaluate the bowel preparation quality, but the bowel preparation regimens varied among the studies. Two studies [15, 17] used a split dose of 2 L polyethylene glycol (PEG) + ascorbate for bowel preparation, one [16] used a single dose of 3 L PEG, and one [19] used a split dose of 4 L PEG. In the multicenter study [18], 90.3% patients received the 4 L PEG preparation, of which the majority (82.9%) adopted a split-dose regimen, and other patents received 2 L PEG or sodium picosulfate + magnesium citrate for bowel preparation. Adequate bowel preparation was defined as a score of ≥ 2 on the BBPS in each segment or a total BBPS score ≥ 6 (Tables 1 and 2). One study [16] had some concerns allocation concealment and other studies had a low risk of bias (Table 3). Since < 10 studies were included, publication bias was not evaluated.

**Table 1:** Characteristics of included studies

Study	Country	Study period	Inclusion age criteria	Indication for colonoscopy	Bowel preparation regimen	Duration of low-residue diet		Definition of adequate bowel preparation
						Single-day group	Multi-day group	
Gimeno-García 2019	Spain	2017.12-2018.03	≥ 18	Screening, surveillance, diagnostic	2 L PEG + ascorbate, split-dose	1 day	3 days	BBPS score ≥ 2 in each segment
Jiao 2020	China	2018.05-2019.03	18–80	Screening, diagnostic	3 L PEG, single dose	1 day	2 days	Total BBPS score ≥ 6
Machlab 2021	Spain	2018.12-2020.01	50–69	Positive results on FIT	2 L PEG + ascorbate, split-dose	1 day	3 days	BBPS score ≥ 2 in each segment
Scaglione 2023	Italy	NA	≥ 18	Screening, surveillance, diagnostic	2 L PEG + bisacodyl, 2 L PEG + ascorbate, 4 L PEG, SPMC, split-dose or single dose	1 day	3 days	BBPS score ≥ 2 in each segment
Taveira 2019	Portugal	2017–2018	NA	Screening, surveillance, diagnostic	4 L PEG, split-dose	1 day	3 days	BBPS score ≥ 2 in each segment

BBPS, Boston Bowel Preparation Scale; FIT, fecal immunochemical test; NA, not available; PEG, polyethylene glycol; SPMC, sodium picosulfate + magnesium citrate

**Table 2:** Characteristics of patients from included studies

Study	Sample size		Average age, mean ± SD		Male, *n* (%)	
	Single-day group	Multi-day group	Single-day group	Multi-day group	Single-day group	Multi-day group
Gimeno-García 2019	196	194	59.7 ± 14.6	60.2 ± 14.3	96 (49.0)	103 (53.1)
Jiao 2020	161	160	48.17 ± 15.44	47.03 ± 13.79	56 (34.78)	70 (43.75)
Machlab 2021	420	416	58.9 ± 5.4	59.3 ± 5.5	234(55.7)	242(58.2)
Scaglione 2023	143	146	60.2 ± 12.4	60.3 ± 13.5	68(47.6)	83(56.8)
Taveira 2019	206	206	67 (16)*	66 (14)*	138(67)	140 (68)

NA, not available; SD, standard deviation.

* Values are showed as median (interquartile range).

**Table 3:** Risk of bias assessment of the included studies using Cochrane Risk-of-Bias 2 tool

Study	Bias arising from the randomization process	Bias due to deviations from intended interventions	Bias due to missing outcome data	Bias in measurement of the outcome	Bias in selection of the reported result	Overall bias
Gimeno-García 2019	Low risk of bias	Low risk of bias	Low risk of bias	Low risk of bias	Low risk of bias	Low risk of bias
Jiao 2020	Some concerns	Low risk of bias	Low risk of bias	Low risk of bias	Low risk of bias	Some concerns
Machlab 2021	Low risk of bias	Low risk of bias	Low risk of bias	Low risk of bias	Low risk of bias	Low risk of bias
Scaglione 2023	Low risk of bias	Low risk of bias	Low risk of bias	Low risk of bias	Low risk of bias	Low risk of bias
Taveira 2019	Low risk of bias	Low risk of bias	Low risk of bias	Low risk of bias	Low risk of bias	Low risk of bias

### Primary outcomes

**Fig. 2:**
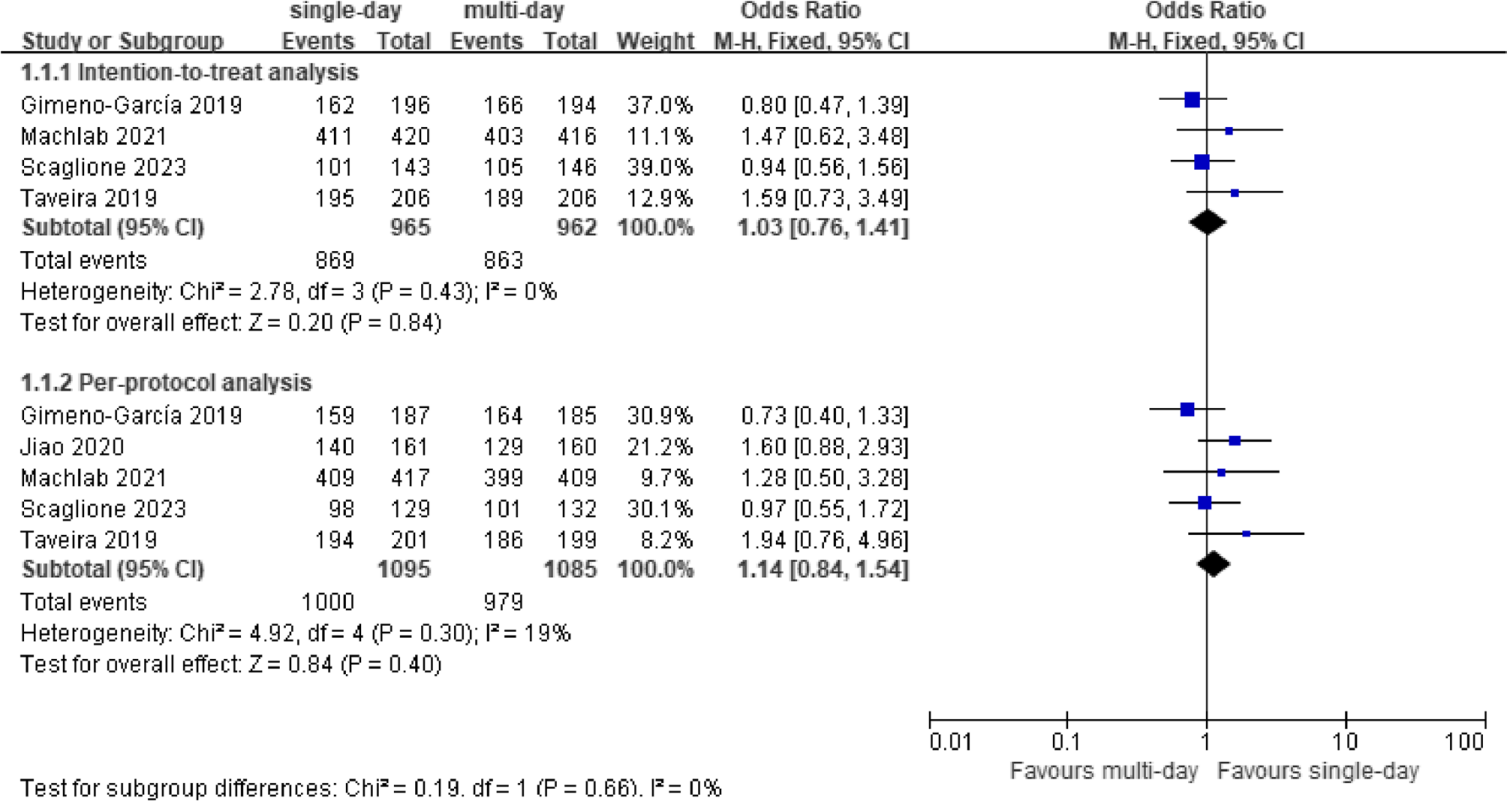
Forest plot comparing rate of adequate bowel preparation

All five studies included in the meta-analysis compared the effectiveness of single-day LRD versus multi-day LRD in bowel preparation quality. The heterogeneity test showed no significant heterogeneity, and a fixed effect model was used. In the ITT population, the adequate bowel preparation rates were 90.1% and 89.7% in the single-day and multi-day LRD groups, respectively (pooled OR = 1.03, 95% CI: 0.75–1.41, *p* = 0.85). In the PP population, the adequate bowel preparation rates were 91.3% and 90.2% in the single-day and multi-day LRD groups, respectively (pooled OR = 1.15, 95% CI: 0.81–1.62, *p* = 0.43). These results suggest no significant difference in the bowel preparation quality between the single-day and the multi-day LRD groups (Fig. 2). The sensitivity analysis suggested that after excluding any study, no significant difference in the OR of the adequate bowel preparation rate was found.

### Secondary outcomes

**Fig. 3:**
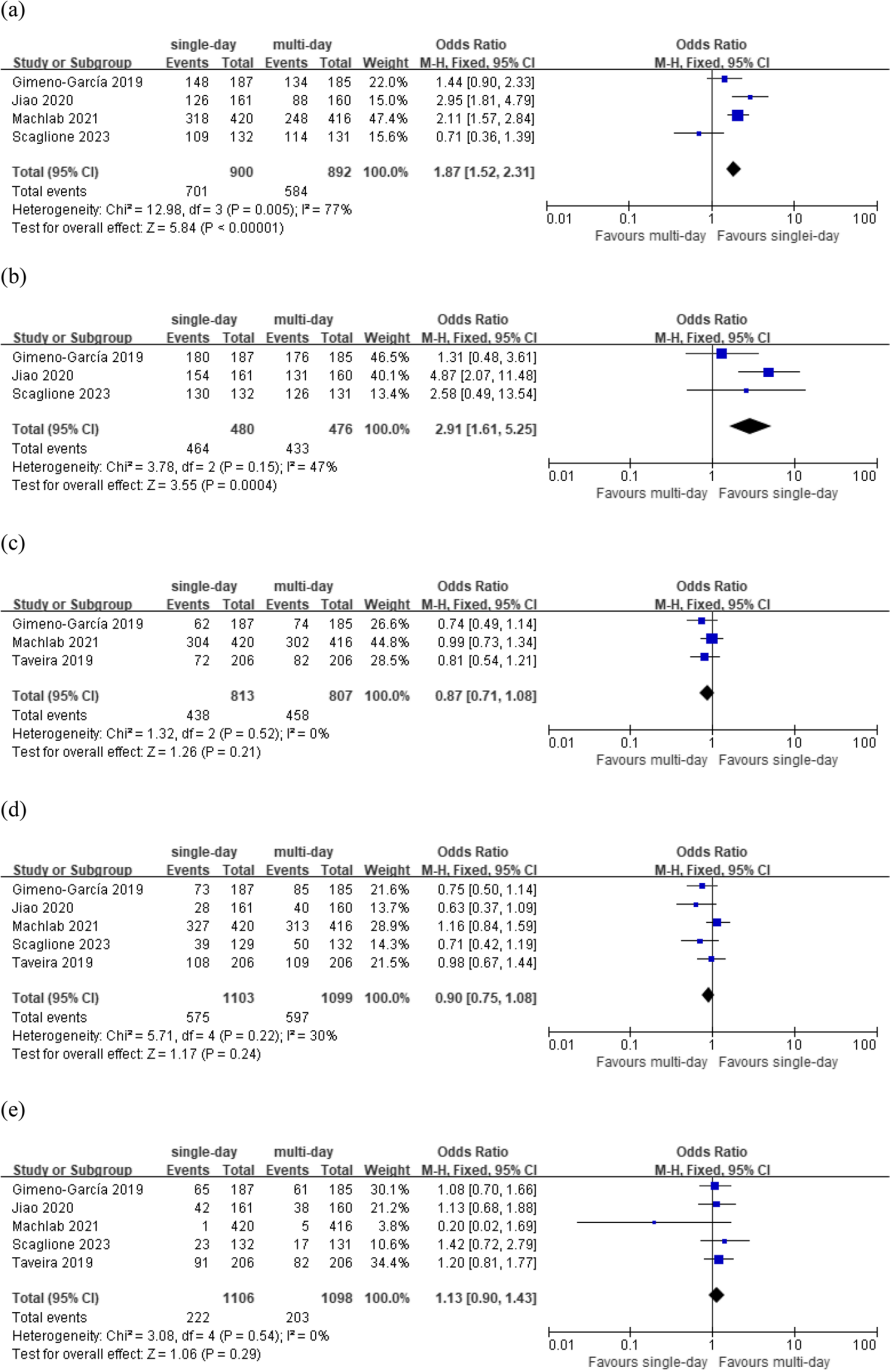
Forest plot comparing **A** patient tolerance, **B** willingness to repeat the same dietary restriction, **C **ADR, **D** PDR, **E** adverse events or incidents

Four of the included studies [15–18] assessed patient tolerance (pooled OR = 1.67, 95% CI: 1.03–2.71, *p* < 0.05) and found that tolerance was significantly higher in the single-day LRD group than in the multi-day LRD group (Fig. 3a). Three studies [15, 16, 18] reported the willingness to repeat the same dietary restriction, and the willingness was significantly higher in the single-day LRD group than in the multi-day LRD group (pooled OR = 2.64, 95% CI: 1.09–6.38, *p* < 0.05, Fig. 3b). There was no significant difference in ADR, PDR, or adverse events or incidents between the two groups (Fig. 3c, d, e). Three studies [15, 16, 19] evaluated hunger sensation of patients. Hunger was one of the most frequent incidents [15], with no statistically significant difference in hunger‑comfort scale [16] or hunger incidence rate [19] between the two groups. Besides, there was no significant difference in overall patient satisfaction between the two groups (pooled OR = 1.35, 95% CI: 0.84–2.16, *p* = 0.22, Supplementary Figure).

(a)

## Discussion

This meta-analysis included five randomized controlled trials comparing single-day and multi-day LRDs for colonoscopy bowel preparation. Our results showed no significant differences in bowel preparation quality, ADR, and PDR between the single-day and multi-day LRD groups, but patient tolerance and willingness to repeat the same dietary restrictions were higher in the single-day LRD group.

Dietary restriction is a crucial step in bowel preparation for colonoscopy. Traditionally, a pre-endoscopic CLD had been recommended before colonoscopy [20]. Several recent meta-analyses of randomized controlled trials have demonstrated that a LRD before colonoscopy is effective as a CLD for bowel preparation quality, but a LRD was associated with higher patient tolerability and willingness to repeat bowel preparation than a CLD [7, 21, 22]. Therefore, some guidelines suggested that a CLD prior to colonoscopy could be replaced with a LRD [8]. However, there is no meta-analysis on the restriction period of LRD. Our study confirmed that a single-day LRD was adequate and tolerable for dietary restriction in colonoscopy bowel preparation.

Systematic reviews or meta-analyses can systematically collect, integrate, and analyze the results of multiple independent studies, thereby improving the reliability of the overall research conclusion [23]. The five randomized controlled trials included in the present meta-analysis had clear inclusion and exclusion criteria for research objectives and consistent intervention methods, primary outcomes, and measurement methods. The studies were of high methodological quality, and the heterogeneity test showed that there was no significant heterogeneity among the studies.

Our findings confirm and extend the conclusions of the recent meta-analysis by Putri et al. [12]. Consistent with their results, we found no statistically significant difference in the quality of bowel cleansing between single-day and multi-day LRD regimens, reinforcing the notion that a shorter diet duration is not inferior in terms of efficacy. More importantly, our analysis moves beyond the question of non-inferiority to address critical aspects of patient experience. We demonstrated that the single-day LRD regimen was associated with significantly better patient tolerability and a higher willingness to repeat the preparation. This is a pivotal finding that Putri et al.‘s work did not capture. While their study answered ‘Which diet cleans better?’, our study answers the subsequent and equally important question: ‘Which diet will patients be more likely to complete satisfactorily?’ The enhanced tolerability of the single-day regimen is not merely a matter of patient convenience; it has direct clinical implications. Improved tolerability likely leads to higher adherence rates, which in turn minimizes the risk of preparation failures and repeat procedures. This paradigm shift from a purely efficacy-focused view to a combined efficacy-tolerability framework is essential for developing future guidelines that are not only evidence-based but also patient-centered.

High-fiber intake prior to colonoscopy was an independent risk factor for inadequate bowel preparation [24]. Patients who had other risk factors for inadequate bowel preparation required other rescue measures to improve bowel preparation, such as increasing polyethylene glycol dosage, eating a low-residue diet for 3 days before endoscopy, and using gastrointestinal motility drugs [25, 26]. Yeh et al.. reported a retrospective cross-sectional study and concluded that a 1-day LRD led to bowel preparation similar to that achieved through a 3-day LRD regardless of the type of cleansing agent or the use of supplemental laxatives [27]. Nevertheless, a post hoc analysis of a randomized controlled trial showed that 3-day LRD is not superior to 1-day LRD in patients with risk factors for inadequate bowel cleansing [28]. A prospective randomized pilot study showed the quality of colon cleanliness achieved with one-day bowel preparation was equivalent to that of the standard two-day schedule in patients undergoing colon capsule endoscopy [29]. Compared to single-day LRD, multi-day LRD requires more preparation time, thereby imposing more restrictions on the patient’s diet and lifestyle and affecting tolerance and compliance with bowel preparation. Our study also showed a higher dietary restriction tolerance and willingness to repeat the same restrictions among patients in the single-day LRD group compared to the multi-day LRD group.

Our study had some limitations. First, this systematic review was not registered in PROSPERO due to the study being completed prior to the journal’s requirement. All methods were pre-specified and adhered to PRISMA guidelines to minimize reporting bias. Second, the indications for colonoscopy in most of the included studies were limited to screening, surveillance, and diagnosis. One study^16^ involved participants from an early colorectal cancer detection program aged 50–69 years, with positive fecal immunochemical test results. This study had higher ADR and PDR than other studies, which may increase the heterogeneity between studies. Third, although all studies used BBPS to evaluate the bowel preparation quality, and a score of ≥ 2 in each segment was considered adequate bowel preparation, the bowel preparation regimens were not consistent throughout the studies. Three studies were split dose, one study was a single dose, and one was a split dose or same-day dose based on the scheduled time of colonoscopy. Fourth, the number of included studies was small, and more randomized controlled studies are needed to validate our results.

We initially planned to employ alternative synthesis methods such as vote counting for outcomes unsuitable for meta-analysis. However, upon detailed extraction, we found that the reporting of hunger was highly inconsistent across studies. Hunger was measured in such radically different ways that grouping them for any comparative count would be misleading. Consequently, a narrative synthesis was deemed the only appropriate method to fairly represent the data without introducing misleading or arbitrary interpretations. We have structured this narrative synthesis to transparently present the findings from each study and to discuss the possible explanations for the observed variations.

## Conclusion

Compared with a multi-day LRD, a single-day LRD before colonoscopy is associated with higher patient tolerance for bowel preparation and willingness to perform similar dietary restrictions again; moreover, there was no significant difference in the bowel preparation quality between the two groups.

## Supplementary Information

Supplementary Material 1.

## Data Availability

All data relevant to the study are included in the article. Extracted data are available from the corresponding author on reasonable request.
