# Supplementary material for: Effect of single-day versus multi-day low-residue diet on colonoscopy bowel preparation: a systematic review and meta -analysis
Source: BMC Gastroenterol. 2025 Sep 26;25:648. doi: 10.1186/s12876-025-04261-8 (PMC12465615; doi:10.1186/s12876-025-04261-8)
Supplement: Supplementary file 1 — Supplementary Material 1. [file 12876_2025_4261_MOESM1_ESM.docx]

Search Strategy

Online English databases

PubMed, Web of Science, Cochrane Library, and Embase

Search terms

(single day OR 1 day OR one day) AND (low residue diet OR low fibre diet OR low fiber diet OR fiber free diet OR fibre free diet OR dietary restriction OR diet liberalization) AND (colonoscopy OR bowel preparation OR colon cleansing)

Filters and limits used

None

Supplementary Table. The tabular summary of the low-residue diet protocols

| Study | Low-residue diet protocols |
| --- | --- |
| Gimeno-García 2019 | BREAKFAST:  - 200 ml of milk or 2 skimmed yoghurts.  - 40 g of white bread or 30 g of corn cereals or 30 g of corn/milled flour or 30 g of white bread toast.  - 1 slice of turkey breast or ham or skimmed cheese spread.  SNACK:  - 40 g of white bread or 30 g of toast.  - 40 g of skimmed tender white cheese or natural canned tuna or ham or turkey.  LUNCH:  - 60 g of white rice or 60 g of white pasta or 100 g of peeled potatoes or 60 g of cooked or boiled couscous without sauces or dressings.  - 100 g of lean meats or 150 g of boiled or grilled white fish or 150 g of egg or 80 g of soft white skimmed cheese.  - 1 skimmed yogurt.  SNACK:  - 200 ml of milk or 2 skimmed yoghurts.  - 20 g of crackers.  DINNER:  - 60 g of white rice or 60 g of white pasta or 100 g of peeled potatoes or 60 g of cooked or boiled couscous without sauces or dressings.  - 100 g of lean meats or 150 g of grilled white fish stew or 150 g of egg or 80 gr of tender white cheese skimmed.  - 1 low-fat yogurt.  The day before the examination, you can only drink water, tea or chamomile for dinner. |
| Jiao 2020 | Low residue foods refer to foods that have a low fiber content, including rice porridge, noodles, taro, bread, tofu, Chinese steamed eggs, chicken, some peeled and cored fruits, and cooked vegetables (such as apples and carrots). Patients were told to avoid eating vegetables, fruits, and whole grains. |
| Machlab 2021 | Dietary instructions were designed by an endocrinologist specialized in nutrition. All the subjects received a logbook and were requested to register the diet along 3 days before colonoscopy.  The specific dietary protocols were not available. |
| Scaglione 2023 | 3-DAYS LOW FIBERS DIET FOR COLONOSCOPY  Day 3 and day 2 before colonoscopy  BREAKFAST (8-9 a.m.), LUNCH (1-2 p.m.) AND DINNER (8-9 p.m.)  Allowed Foods: pasta or rice (not whole), meat, fish, milk, eggs, cheese, ham and cold cuts, bread (not brown or rye bread), potatoes  Forbidden foods: vegetables, legumes, fruits  Day 1 before colonoscopy  BREAKFAST (8-9 a.m.) AND LUNCH (1-2 p.m.)  Allowed Foods: pasta or rice (not whole), meat, fish, milk, eggs, cheese, ham and cold cuts, bread (not brown or rye bread), potatoes  Forbidden foods: vegetables, legumes, fruits  DINNER (8-9 p.m.)  Allowed foods: clear broth, fruit juice without pulp, tea, honey and Camomille  1-DAY LOW FIBERS DIET FOR COLONOSCOPY  Day 1 before colonoscopy  BREAKFAST (8-9 a.m.) AND LUNCH (1-2 p.m.)  Allowed Foods: pasta or rice (not whole), meat, fish, milk, eggs, cheese, ham and cold cuts, bread (not brown or rye bread), potatoes  Forbidden foods: vegetables, legumes, fruits  DINNER (8-9 p.m.)  Allowed foods: clear broth, fruit juice without pulp, tea, honey and Camomille |
| Taveira 2019 | Day -3 and day -2 before colonoscopy (for the 3-day low fibre diet)  BREAKFAST  What you can eat: lean or medium-fat milk, tea (lime, lemon, chamomile, etc.), white bread with a small quart of butter or margarine, ham, jelly or fresh cheese, wafers (e.g. water and salt cookie), dried biscuits without fibre; cakes like sponge cake, natural, flavour, bifidus or liquid yogurt, coffee  What you cannot eat: fat or soy milk / black tea / green tea, corn, whole wheat or mixed bread, cereals, cakes with cream, chocolates, whole wheat wafers, fruit or cereals yogurt, fruit (kiwis, grapes, orange, ...)  LUNCH AND DINNER  What you can eat: white bread, rice, potatoes, pasta, boiled or poached egg, lean fish (e.g. white fish) cooked / grilled on the electric grill, lean meats (chicken, ham, rabbit, turkey), cooked and grilled on the electric grill, vegetables, (e.g.: cucumbers, kale, lettuce, turnips, broccoli, peppers, onions, carrots, tomatoes, greens, watercress, spinach, etc.) and leguminous (peas, beans, etc.), white soups (rice, potato and pasta), chicken soup, pineapple, lemon or peach jelly, non-carbonated soft drinks (e.g. iced tea, ...)  What you cannot eat: corn, whole wheat or mixed bread, cereals, meat / fish, fried, cooked, baked in the oven, sausages, fruit (kiwis, grapes, orange, ...), carbonated drinks, nectars / fruit juices or foods that are not light-coloured  ATTENTION**  - Food should be well cooked and well chewed.  - Meals should be 5 to 7 per day and in small amounts.  - Drink plenty of water (at least 1.5 litres per day).  - Cannot drink coffee the day before and the day of the exam.  Day -1 before colonoscopy (for the 3-day and 1-day low fibre diet)**  BREAKFAST  Lean or medium-fat milk, tea, white bread with butter, ham, jelly or cheese in small quantity, biscuits / wafers without seeds or fibres, natural, flavour, bifidus or liquid yogurt  LUNCH  Chicken Soup, pineapple, lemon or peach jelly  After 4 p.m.  You can only drink water, tea, non-carbonated soft drinks and light-coloured jelly. |

Supplementary Figure


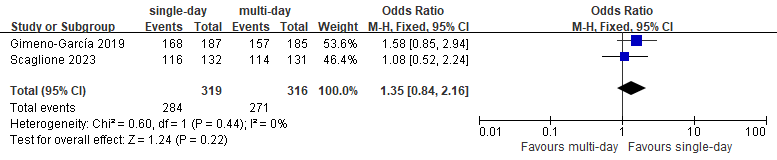


Supplementary Figure. Forest plot comparing overall patient satisfaction.
